# Natural Variation in the Strength and Direction of Male Mating Preferences for Female Pheromones in *Drosophila melanogaster*

**DOI:** 10.1371/journal.pone.0087509

**Published:** 2014-01-28

**Authors:** Alison Pischedda, Michael P. Shahandeh, Wesley G. Cochrane, Veronica A. Cochrane, Thomas L. Turner

**Affiliations:** 1 Department of Ecology, Evolution and Marine Biology, University of California Santa Barbara, Santa Barbara, California, United States of America; 2 Department of Cell and Developmental Biology, Oregon Health and Science University, Portland, Oregon, United States of America; University of Arkansas, United States of America

## Abstract

Many animal species communicate using chemical signals. In *Drosophila*, cuticular hydrocarbons (CHCs) are involved in species and sexual identification, and have long been thought to act as stimulatory pheromones as well. However, a previous study reported that *D. melanogaster* males were more attracted to females that were lacking CHCs. This surprising result is consistent with several evolutionary hypotheses but is at odds with other work demonstrating that female CHCs are attractive to males. Here, we investigated natural variation in male preferences for female pheromones using transgenic flies that cannot produce CHCs. By perfuming females with CHCs and performing mate choice tests, we found that some male genotypes prefer females with pheromones, some have no apparent preference, and at least one male genotype prefers females without pheromones. This variation provides an excellent opportunity to further investigate the mechanistic causes and evolutionary implications of divergent pheromone preferences in *D. melanogaster* males.

## Introduction

Chemical signaling is an important form of communication for many animal species [1]. For most insects, chemical communication occurs primarily via hydrocarbons that cover the epicuticle [2]. In *Drosophila*, cuticular hydrocarbons (CHCs) are critical communication signals both within and between species. CHCs are involved in species identification, and play key roles in sexual isolation between *Drosophila* species [3]–[6] and incipient species [7], [8]. CHCs also act as pheromones within species, communicating sexual identity [3], [9], female mating status [10]–[13] and age [14].

Despite considerable investigation, the roles that CHCs play in mating preferences in *D. melanogaster* are still unclear. A previous study by Billeter et al. [3] reported a surprising discovery: *D. melanogaster* males preferred to mate with virgin females lacking CHCs compared to those normally expressing CHCs. Although counter-intuitive, this finding is consistent with several evolutionary hypotheses. It has been previously suggested that inhibitory pheromones may be advantageous to females because they slow mating attempts by males, allowing females time to assess male quality and/or species identity [3]. There is also evidence that pheromones are involved in sexual conflict by altering the attractiveness of mated females [10], [11], [13]. During mating, males transfer the ejaculatory pheromone *cis*-vaccenyl acetate (cVA) to females to deter future mating attempts by other males [15], [16]. Although cVA is a strong male courtship deterrent, the female-specific CHC 7,11-heptacosadiene (7,11-HD) acts as an attractant to mitigate the aversive effect of cVA [3]. Thus, an alternative hypothesis is that the primary role of female CHCs is to inhibit the aversive pheromones transferred by males during mating, with a reduction in the attractiveness of virgin females occurring as a side effect. Finally, it is possible that virgin female CHCs that are slightly unattractive to conspecific males are favored by selection because of their strongly inhibitory effects on males of the sympatric *D. simulans* [3]–[5], as interspecies matings produce no fertile offspring and persistent harassment by undesirable heterospecific males could be very costly for females [17].

Although it seems plausible that selection could favor female pheromones that are unattractive to males of their own species, this finding is at odds with previous research. Male mating preferences for female CHCs have been documented in several *Drosophila* species, including *D. serrata* [18], [19], *D. mojavensis* [20], D. *virilis* and *D. lummei* [21]. Similarly, multiple studies have reported that female CHCs stimulate male courtship in *D. melanogaster* [5], [22]–[27] and that males prefer to mate with females expressing CHCs when given the choice [28]. These studies used different methods than the study by Billeter et al. [3], in which CHCs were eliminated using transgenic manipulations that destroyed the oenocytes (the cells required for CHC production). It is unclear what role, if any, methodology plays in these conflicting results. As the role of CHCs is fundamental to understanding the molecular and neurological basis of courtship behavior and the specific traits involved in sexual selection and sexual conflict in *Drosophila*, we have attempted to address these discrepancies here. Our goals were to: *i*) replicate the results of Billeter et al. [3] using their methods, *ii*) determine if their findings were a result of inadequate controls, and *iii*) test for natural variation in the strength and direction of male preferences for female CHCs in *D. melanogaster*. Though we are able to replicate the results of Billeter et al. [3], we find that their method may imperfectly control for the effects of genetic manipulation, and that there is considerable genetic variation among *D. melanogaster* males in the strength and direction of their preferences for female CHCs. This variation provides an excellent opportunity to investigate the causes and consequences of divergent pheromone preferences at an incipient stage.

## Methods

### *D. melanogaster* stocks and maintenance

Unless otherwise stated, all fly strains were maintained in 20 mm vials on standard cornmeal/molasses/yeast medium on a 12 h: 12 h light/dark cycle at 25°C. Under these conditions, non-overlapping two-week lifecycles were established as follows: male and female adult flies were transferred into fresh vials containing food media for 1–3 days before being discarded. 14 days later (after all progeny had eclosed), flies were transferred into fresh food vials for 1–3 days to begin the next generation.

We created flies without oenocytes (“oe^−^ flies”) using the protocol described by Billeter et al. [3]. Briefly, oe^−^ flies were created by crossing “+ : *UAS-StingerII*, *UAS-hid/CyO*; +” to “+ : *PromE(800)-Gal4, tubP:Gal80^ts^*; +”. Progeny were reared at 18°C and virgin oe^−^ males and females were collected within 6 h of eclosion under light CO_2_ anesthetization at room temperature. Adults were held at 25°C for 24 h before being subjected to three consecutive overnight heat treatments (12 h at 30°C). Flies were returned to 25°C for 12 h between heat treatments. This heat treatment was necessary to ensure the destruction of the oenocytes. Before experiments began, the effectiveness of this treatment was verified using a fluorescence microscope to ensure that ∼100 oe^−^ females and ∼50 oe^−^ males lacked GFP-labeled oenocytes. We then screened a subset of oe^−^ flies (∼30) for each experiment (or replicate) to ensure the heat shock protocol had effectively ablated oenocytes. Flies were allowed to recover from this heat shock treatment for 24 h at 25°C before experiments, which were conducted when the flies were 6 days old.

We also created “control” flies as per Billeter et al. [3] by crossing “+ : *PromE(800)-Gal4, tubP:Gal80^ts^*; +” to “+ : *UAS-StingerII*; +”. Progeny from this cross were collected and treated identically to oe^−^ flies but did not experience oenocyte destruction during the heat treatment, allowing them to express CHCs. Though Billeter et al. referred to these flies as “controls”, will refer to them as “oe^+^” to differentiate them from our additional control: oe^−^ females that were perfumed with CHCs (see below). All stocks used to make oe^−^ and oe^+^ flies were provided by J. D. Levine (University of Toronto at Mississauga), as was the *D. simulans* stock used below.

For our perfuming protocol, we used *D. melanogaster* females from an outbred, laboratory-adapted population that were homozygous for the recessive *white* marker, causing them to be white-eyed (distinguishable from oe^−^ females, which appear wild-type). We received this *white* population from W. R. Rice (University of California, Santa Barbara).

To test for genetic variation in male attraction to female CHCs, we used the wild-type Canton-S strain of *D. melanogaster* and 11 of the 15 inbred “founder” lines of the Drosophila Synthetic Population Resource [29], which were originally collected from diverse locations: BOG1 (Bogota, Columbia), KSA2 (Kariba Dam, South Africa), VAG1 (Athens, Greece), wildB5 (Red Top Mountain, Georgia, USA), T.7 (Ken-ting, Taiwan), BER1 (Bermuda), CA1 (Capetown, South Africa), QI2 (Israel), RVC3 (Riverside, California, USA), T.1 (Ica, Peru), and T.4 (Kuala Lumpur, Malaysia). Three founder lines (BS1, Sam *ry* ^506^ and T.0) were excluded because they are weak stocks with males that were often unsuccessful at mating, and the fourth founder line was not included because it is a replicate of Canton-S. Canton-S was obtained from the Bloomington Drosophila Stock Center in 2011 and the founder lines were obtained from S. J. Macdonald (University of Kansas) in November 2011. All lines have since been maintained on two-week cycles as described above.

We also used the “allRAL” population described in detail elsewhere [30] to test the preference of outbred males. Briefly, this population was initiated by crossing males and females from 173 RAL inbred lines created in the lab of T. F. C. Mackay [31], [32]. These lines were established using *D. melanogaster* collected in Raleigh, North Carolina, and by crossing them together we can approximately recreate the outbred population from this area. At the time of our experiments, this population had undergone 43 generations of outbreeding.

### Testing for male-male courtship using oe^−^ males

We collected oe^−^ and oe^+^ males as virgins and transferred them individually into small vials containing food media for the heat treatment described above. Courtship observations were conducted within 2 h of lights-on, and used standard vials (20 mm in diameter) containing a small amount of food media with a foam plug pushed into the vial to allow for an interaction space 20 mm in diameter by ∼5 mm in height. Pairs of males were gently aspirated into the vials to create the following three treatments: 2 oe^−^ males, 2 oe^+^ males, and 1 oe^−^ male with 1 oe^+^ male. Aspirator tips were changed between oe^−^ and oe^+^ males to prevent CHC contamination.

Observers who were blind to the treatments watched male pairs for 30 min and recorded courtship behaviors (following, wing extensions, attempted copulations, and “head-to-head” interactions) in 1-minute intervals. We calculated a courtship index for each pair as the proportion of time spent courting by either male during a 10 minute period that began when courtship was initiated [3]. We surveyed 19–20 pairs of males per treatment.

### Cuticular hydrocarbon transfer to oe^−^ females

We collected donor females from the *white* population as virgins and held them in groups of 20 in vials containing food media for 6–7 days before the experiments began. At this point, we transferred 60 *white* females into an empty vial (containing no food medium) and added 10 oe^−^ females. We transferred CHCs from the *white* females to the oe^−^ females by subjecting them to three medium vortex pulses lasting 20 seconds, with a 20 second break between pulses (method adapted from [33]). CHC transfer between individuals by physical contact or “rubbing” has been widely used to study CHC preferences in *Drosophila* [5], [8], [13], [33], [34], and a nearly identical version of this perfuming protocol has been used previously to successfully transfer CHCs to oe^−^ females [35].

To control for the perfuming procedure, we “sham-perfumed” oe^−^ females by transferring 70 oe^−^ females into an empty vial and vortexing them as described above. 70 oe^−^ females were used to keep the number of individuals in each vial consistent with the perfumed treatment (which had 10 oe^−^ and 60 *white* females in each vial). This ensures that females with and without CHCs were held under identical conditions before the experiments began.

### Validation of CHC transfer using *D. simulans* male courtship

We observed the courtship behavior of *D. simulans* males when paired with 5 different types of females: *D. simulans* females, *D. melanogaster* oe^+^ females (expressing CHCs), oe^−^ females (lacking CHCs), perfumed oe^−^ females, and sham-perfumed oe^−^ females. All experimental flies were collected as virgins under light CO_2_ anesthetization and held in vials containing food media for 6 days (females were held in groups of 10 and males were held in groups of 20). During this time, *D. simulans* males and females were held at 25°C, and all oe^−^ and oe^+^ females were subjected to the heat treatment described above.

We performed courtship observations within the first 4 h after lights-on using 35 mm×10 mm plastic Petri dishes with a small amount of food media coating the bottom. Perfumed and sham-perfumed oe^−^ females were vortexed as described above and transferred in groups of 10 to vials containing food media to recover for 1 h. At this time, we gently aspirated individual pairs of flies (*D. simulans* males with one of the five different female types) into the Petri dishes. Aspirator tips were changed between different fly types to avoid CHC contamination.

Observers who were blind to the treatments watched the pairs for 1 h and recorded courtship behaviors (following, wing extensions, and attempted copulations) in 1-minute intervals. We used this information to calculate a courtship index for each male as the proportion of time spent courting over a 10 minute period starting with the initiation of courtship [3]. In total we surveyed 15–30 pairs per treatment.

### Testing male preferences for female CHCs

We first performed mate choice trials using the Canton-S strain to replicate the results of Billeter et al. [3], and then tested for genetic variation in male CHC preferences using the 11 genotypes described above and our outbred allRAL population. All mate choice trials were conducted using the methods described below.

We collected virgin oe^−^ and oe^+^ females under light CO_2_ anesthetization and held them in groups of 10 during the heat treatment described above. We also collected experimental males as virgins under light CO_2_ anesthetization and held them in groups of 20 at 25°C for 6 days. We conducted mate choice trials within 4 h of lights-on using 20 mm vials containing a small amount of food media, with a foam plug pushed into the vial to create a space 20 mm in diameter by ∼5 mm in height. Males and females from the same holding vial were distributed across treatments to avoid pseudoreplication. We observed these vials for 1 h; when a mating occurred, the unmated female was aspirated out of the vial for identification (see below).

For mate choice trials using oe^−^ and oe^+^ females, we gently aspirated one oe^−^ and one oe^+^ female into a vial before adding a single male. Aspirator tips were changed between oe^−^ and oe^+^ females to avoid CHC contamination. We were able to determine the genotype of the unmated female using a dissecting microscope equipped with fluorescent illumination, as the oenocytes of the oe^+^ females are GFP-positive and the oe^−^ females are GFP-negative.

For mate choice trials using perfumed oe^−^ females, we differentiated females from the two treatments by making small holes in either their left or right wings using a dissecting pin. This procedure was completed the day after the females were collected as virgins, before they were subjected to their first heat treatment. There was no additional mortality observed in the oe^−^ females that had their wings modified. To begin the mate choice trials, oe^−^ females were either perfumed or sham-perfumed using the vortex treatment described above. After vortexing, we briefly (∼30 s) anesthetized females using light CO_2_ and set up experimental vials (containing a small amount of food media) with a single perfumed female and a single sham-perfumed female. These females were allowed to recover from the procedure for ∼1 h (but no more than 90 minutes) before experiments began.

For each male genotype, we calculated a preference index to measure the strength of the preference for oe^−^ females [3]. This was calculated as the number of males that mated with oe^−^ (or sham-perfumed) females minus the number of males that mated with oe^+^ (or perfumed) females divided by the total number of trials. This value ranges from −1 to +1, with positive values indicating a preference for females lacking CHCs, and negative values indicating a preference for females with CHCs.

### Cuticular hydrocarbon analysis

We measured cuticular hydrocarbons for virgin oe^−^, oe^+^, perfumed oe^−^ and *white* females that were 6 days old (as in our experiments). For CHC analysis, flies were gently aspirated into individual glass microvials containing 100 µL of hexane supplemented with 10 ng/mL each of octadecane (C18) and hexacosene (C26) as internal standards. Flies soaked in hexane solution for 3 minutes, were then gently agitated with a vortex mixer for 1 minute and finally were removed using fine forceps. CHCs were analyzed using a Shimadzu GC-2014 with Flame Ionization (Shimadzu Scientific Instruments, Columbia, Maryland, USA) fitted with a 15 m×0.25 mm Restek SHRXI-5MS fused silica capillary column with a film thickness of 0.25 µm. Carrier gases were helium and compressed air with a linear velocity flow control mode set at 73.2 cm/s and a column flow rate of 3.0 mL/min. A 2 µL sample of hexane solution was loaded into the GC using a splitless injection mode. The column temperature was held at 55°C for 2 minutes before being heated to 150°C at a rate of 66.6°C/min, then increased to 280°C at a rate of 7°C/min and was held at 280°C for 8 minutes.

### Data analysis

We compared courtship indices for the *D. simulans* courtship trials and the *D. melanogaster* male-male courtship trials using Wilcoxon tests followed by posthoc analysis with sequential Bonferroni tests [36] to correct for multiple comparisons. We tested for variation among male genotypes in the proportion of oe^−^ females mated (i.e. number of oe^−^/sham-perfumed females mated vs. number of oe^+^/perfumed females mated) using a Chi-square test, and determined whether the preference indices calculated for each male genotype were significantly different from 0 using binomial tests (followed by sequential Bonferroni tests to correct for multiple tests across genotypes [36]). We then used a 2-tailed sign test to determine whether the majority of genotypes had positive or negative preference indices. Finally, we tested for a correlation between the preference indices obtained using perfumed females and oe^+^ females with a Pearson's correlation test (after first ensuring both distributions were normal). All analyses were completed using JMP 9.

## Results

### Replication of previous findings and validation of CHC transfer by “perfuming”

Billeter et al. [3] produced a useful and novel tool: a method to create flies lacking oenocytes (“oe^−^” flies) and therefore lacking CHCs. These transgenic flies were generously shared with us, and we first used them to replicate previous findings. We successfully replicated the results of Billeter et al. [3] regarding CHCs and male-male courtship. Combining two oe^−^ males or one oe^−^ with one oe^+^ male induced significantly higher courtship indices (CIs) than the combination of two oe^+^ males (mean CIs ± standard error: oe^−^ x oe^−^  = 0.67±0.07, oe^−^ x oe^+^  = 0.64±0.06, oe^+^ x oe^+^ = 0.26±0.04; Wilcoxon test: full model χ^2^ = 23.55, df = 2, p<0.0001). Likewise, the combination of two oe^−^ males spent a higher proportion of time (in minutes) engaged in “head-to-head” courtship interactions than either of the other two combinations (mean proportion time ± standard error: oe^−^ x oe^−^ = 0.37±0.05, oe^−^ x oe^+^ = 0.02±0.01, oe^+^ x oe^+^ = 0.005±0.005; Wilcoxon test: full model χ^2^ = 35.28, df = 2, p<0.0001).

**Figure 1:**
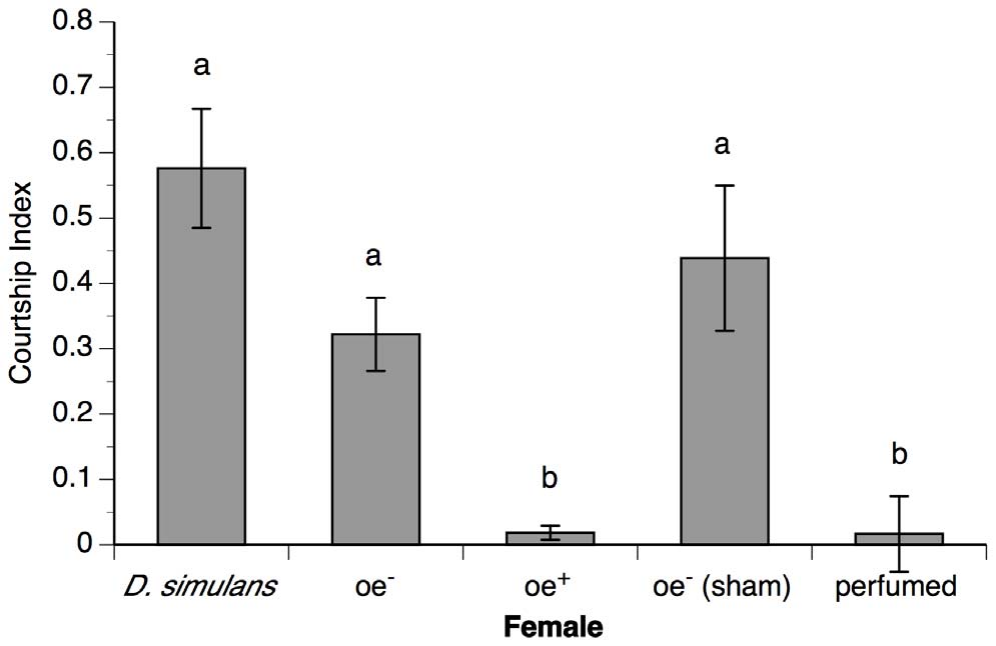
Preferences of *D. simulans* males. Mean courtship indices for *D. simulans* males when paired with: *D. simulans* females, oe^−^ *D. melanogaster* females (lacking CHCs), oe^+^ *D. melanogaster* females (expressing CHCs), sham-perfumed oe^−^ females and oe^−^ females perfumed with *D. melanogaster* female CHCs. Error bars indicate standard errors, and columns labeled with different letters are significantly different from one another (pairwise Wilcoxon tests followed by sequential Bonferroni adjustment, p<0.05). N = 15–30.

We also replicated earlier findings showing that *D. simulans* males courted oe^−^ females more intensely than oe^+^ females (Figure 1; Wilcoxon test: full model χ^2^ = 41.09, df = 4, p<0.0001). Importantly, we found no significant difference in the courtship index of *D. simulans* males when paired with oe^+^ *D. melanogaster* females (with normal CHC production) and when paired with our perfumed oe^−^ females, demonstrating that our perfuming technique was successful at transferring CHCs to oe^−^ females. We also saw that *D. simulans* males courted our sham-perfumed oe^−^ females just as vigorously as regular (not vortexed) oe^−^ females (Figure 1), indicating that our vortexing treatment does not negatively affect the attractiveness of oe^−^ females.

We further validated our perfuming protocol by analyzing CHC profiles of oe^−^, oe^+^, perfumed and *white* females using gas chromatography (Figure S1). These results indicate that we were effective at transferring CHCs from *white* females to oe^−^ females, but also that the CHC profile of perfumed females may differ in concentration and composition from that of the oe^+^ females.

### Genetic variation in male preferences for female CHCs

**Figure 2:**
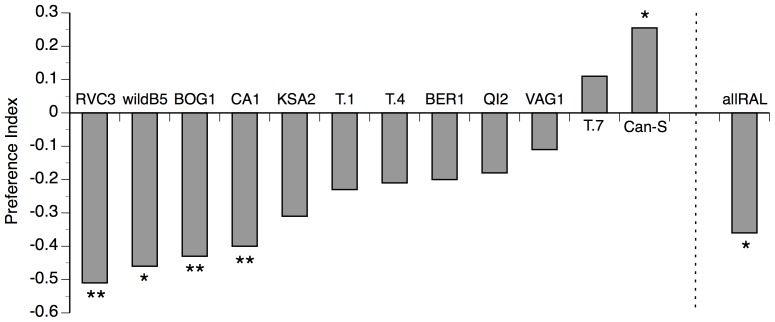
Preference variation in *D. melanogaster* males. Preference indices for *D. melanogaster* males from 12 inbred genotypes and one outbred population (allRAL) when allowed to choose between perfumed oe^−^ females and sham-perfumed oe^−^ females (lacking CHCs). The preference index is the relative advantage of oe^−^ females over perfumed females, such that positive values indicate a preference for females lacking CHCs. Asterisks above the columns show preference indices that are significantly different from 0 (binomial tests: * p<0.05; ** p<0.01 and significant after sequential Bonferroni adjustment). N (Canton-S)  = 94, all other n = 26–60.

We replicated the original finding of Billeter et al. [3], demonstrating that males of the Canton-S strain of *D. melanogaster* significantly prefer oe^−^ females to oe^+^ females (preference index  = 0.47; binomial test: n = 71, p = 0.0001; Figure S2). This preference for females lacking CHCs was consistent (although not as strong), when we tested Canton-S male preference for perfumed vs. sham-perfumed oe^−^ females (preference index  = 0.26; binomial test: n = 94, p = 0.017; Figure 2).

When we expanded the experiment to include 11 natural isolates from around the world, we found significant variation in the proportion of males mating with oe^−^ females among the 12 surveyed genotypes (Chi-square test: χ^2^ = 36.9, df = 11, p = 0.0001). In contrast to Canton-S, males from other genotypes demonstrated either no significant preference between perfumed and oe^−^ females (7/11 genotypes), or a strong preference for perfumed females (4/11 genotypes; Figure 2). Although not all isolates had significant preferences in either direction, most genotypes (10/12) had negative preference indices (i.e. preference for females with CHCs; 2-tailed sign test: p = 0.04). This conclusion is consistent with the finding that males from our outbred allRAL population showed a significant preference for perfumed females compared to oe^−^ females (preference index  = −0.36; binomial test: n = 56, p = 0.01; Figure 2).

Although the Canton-S male preference for oe^−^ females was consistent whether we used perfumed females or the oe^+^ control females of Billeter et al. [3], this was not the case for other genotypes. Preference indices were not significantly different from 0 for 10 of the 11 other genotypes when we competed oe^−^ females with oe^+^ females (Figure S2; binomial tests: all n>23 and p>0.12), nor were they for our outbred allRAL population (preference index  = 0.03; binomial test: n = 58, p = 0.9). The only genotype with a significant preference index using this method was T.4 (preference index  = 0.35; binomial test: n = 49, p = 0.02; Figure S2), and the preference was in the opposite direction from that seen using perfumed females (Figure 2). There was no significant correlation between the preference indices when using perfumed females (our control) vs. the oe^+^ control females used by Billeter et al. [3] for these 12 genotypes (Pearson's correlation test: r = 0.45, p = 0.14).

## Discussion

Many published results support a hypothesis that *D. melanogaster* male courtship is induced by female CHCs [5], [22]–[28]. However, Billeter et al. [3] reported that males prefer to mate with females lacking CHCs over females expressing CHCs when given the choice. We attempted to resolve these inconsistencies using the methods of Billeter et al. [3] and tested for natural variation in the strength and direction of male preferences for female CHCs.

We used flies lacking oenocytes, the cells required for CHC production, to successfully replicate three main findings from the study by Billeter et al. [3]. *D. melanogaster* males intensely court oe^−^ males lacking CHCs, demonstrating that these compounds are used in sexual identification. Similarly, *D. simulans* males courted *D. melanogaster* females much more heavily if they lacked CHCs, demonstrating that these pheromones are also used in species identification (Figure 1). Finally, we showed that *D. melanogaster* males from the Canton-S strain preferred to mate with oe^−^ females lacking CHCs when given a choice between oe^−^ and oe^+^ females (Figure S2). It is this last result that we are most interested in, as it suggests that *D. melanogaster* males may indeed prefer conspecific females that lack pheromones.

The three assays above used the “control” oe^+^ males and females designed by Billeter et al. [3]. These flies have genotypes similar to the oe^−^ females but lack the apoptotic *hid* gene responsible for oenocyte ablation. Although similar, the genotypes of the oe^−^ and oe^+^ flies are not the same, and they have at least one significant behavioral difference: oe^−^ males exhibit higher levels of locomotory activity [3]. Male and female locomotory levels are genetically correlated in *D. melanogaster* [37], so it is plausible that oe^−^ females will also exhibit elevated locomotory activity. Because female movement stimulates male courtship [38], differences in locomotory activity between oe^−^ and oe^+^ females could affect the outcome of male mate choice and confound attempts to test for male preferences based on female CHCs alone.

Because of this potential confound, we tested male preferences for female CHCs using oe^−^ females that had been “perfumed” with CHCs by vortexing them with an excess of donor females (compared to “sham-perfumed” oe^−^ females that were vortexed with other oe^−^ females). *D. simulans* males courted our sham-perfumed females much more strongly than our perfumed females (Figure 1), and there was no difference in the amount of courtship directed toward our perfumed oe^−^ females and the oe^+^ *D. melanogaster* females described above. Combined with CHC analysis using gas chromatography (Figure S1), these results indicate that our perfuming was successful at transferring *D. melanogaster* CHCs to oe^−^ females, consistent with previous work [35]. We feel that the perfuming techniques used here and in other studies [34], [35] provide a better control than the oe^+^ flies used by Billeter et al. [3] because they present a choice between individuals that have identical genotypes and life history, differing only in whether or not they have CHCs. This added level of control should allow a more accurate assessment of male preferences for female CHCs within *D. melanogaster*, where relative preferences may be subtle or variable.

While our data indicate that Canton-S males prefer females lacking CHCs, we found substantial variation in the strength and direction of this preference when we surveyed males from 11 additional inbred lines (Figure 2). Out of the 12 total genotypes we tested, only Canton-S demonstrated a preference for females lacking CHCs, with the majority showing preferences in the direction of perfumed females. In addition, males from our outbred allRAL population preferred to mate with perfumed females over oe^−^ females lacking CHCs. Taken together, these results suggest that *D. melanogaster* males in general prefer to mate with females expressing CHCs, but that there can be substantial variation in the strength of this preference, with at least some genotypes having the opposite preference.

We found no significant correlation between male mate preferences using perfumed females (Figure 2) and oe^+^ females (Figure S2), supporting our assertion that the two methods do not measure the same aspects of male mate choice. These inconsistencies may be due to the potential behavioral differences between oe^−^ and oe^+^ females discussed above, but they may also reflect limitations associated with our perfuming technique. The quantity of CHCs transferred to our perfumed flies appears lower than those naturally found on oe^+^ females (Figure S1). If male preferences for female CHCs are dose-dependent, as has been found in *D. mojavensis* [39], then CHCs may be more attractive to *D. melanogaster* males at lower concentrations compared to higher concentrations. Similarly, the CHC profile of the *white* females used for perfuming differs somewhat from that of the oe^+^ females (Figure S1), which might explain our inconsistent results using the two methods. It is worth noting, however, that we found substantial genetic variation in male preferences using either control.

Though we have documented variation in pheromone preferences in *D. melanogaster*, the causes and consequences of this variation remain to be determined. It is possible that females lacking CHCs mimic young, sexually immature females. Female-specific CHCs do not develop until females are sexually mature (reviewed in [40]), and *D. simulans* males court young *D. melanogaster* females more frequently than mature females [41], similar to the result we see using oe^−^ females. *D. melanogaster* males engage in forced copulations with sexually immature females [42], and Canton-S males can sire offspring through these forced copulations [43], [44], so a male preference for females lacking CHCs may reflect an alternate male mating strategy. Instead, variation in male preferences may be cryptic variation rarely expressed in natural populations, revealed only by the inbreeding process and/or a novel environmental context [45]. In either case, this variation may be useful for investigating the molecular and neurological basis of preferences.

Our study raises several interesting questions that warrant further investigation. The males we used for this survey were collected as virgins, before adult CHCs were fully developed. *D. melanogaster* males learn courtship and mating behaviors based on previous experience, and much of this learning is mediated through CHCs and other pheromones [46]–[48]. It is unclear whether the strength and/or direction of male preferences would be changed by previous mating experience, and whether this learning might lessen the genetic variation we see for this trait. Additionally, we perfumed oe^−^ females using females from a single population, but female CHCs vary within and between populations of *D. melanogaster* [49]–[52]. As the male genotypes we surveyed were collected from several diverse locations, it is possible that variation in male mate preferences correspond to female CHC composition within their original populations. Thus, male preferences for perfumed vs. oe^−^ females could change in strength and/or direction depending on the female genotype used for perfuming. Indeed, our study points to the risks associated with extrapolating results from a single genotype to an entire population or species. Canton-S is one of the most frequently studied strains of *D. melanogaster*, but we have demonstrated that it is not necessarily representative of the species as a whole (see also [53]). In addition, had we only surveyed males from our outbred population, we would have missed interesting (and potentially important) variation among male genotypes. While it is not always possible (or practical) to survey both an outbred population and multiple genotypes, we should remain aware that “wild-type” genotypes can vary dramatically in phenotype, and that population averages can mask this variation.

Regardless of the evolutionary reasons behind the genetic variation in this male mating preference, it is dramatic among the 12 genotypes we surveyed. These genotypes are the “founder lines” of the Drosophila Synthetic Population Resource [29], [54], a panel of over 1700 recombinant inbred lines available for high-resolution QTL mapping of specific phenotypes. The variation we see among the founder genotypes suggests that this resource could be used for genetic mapping of male pheromone preference variation. If genetic mapping can be combined with additional experiments testing the impact of male learning and male-female genotype interactions on these preferences, it could greatly enhance our genetic, molecular, neurological and evolutionary understanding of male courtship behavior and mate preferences in *D. melanogaster*.

## Supporting Information

Figure S1 **CHC profiles of individual flies.** Chromatograms are shown for 2 females from each of the following treatments: oe^−^, oe^+^, *white* (the donor females used for perfuming), and oe^−^ females perfumed with the CHCs of *white* females. Internal standards are marked on the chromatograms with a “*”. Note that the y-axis is scaled differently for the *white* females.
(TIFF)

Figure S2 ***D. melanogaster*** **male preferences when paired with oe^−^ and oe^+^ females.** Preference indices for *D. melanogaster* males from 12 inbred genotypes and one outbred population (allRAL) when allowed to choose between oe^+^ females (expressing CHCs) and oe^−^ females (lacking CHCs). The preference index is the relative advantage of oe^−^ females over oe^+^ females, such that positive values indicate a preference for females lacking CHCs. Asterisks above the columns show preference indices that are significantly different from 0 (binomial tests: * p<0.05; ** p = 0.0001 and significant after sequential Bonferroni adjustment). N (Canton-S)  = 71, all other n = 23–54.
(TIFF)
