# Supplementary figures and images for: Natural Variation in the Strength and Direction of Male Mating Preferences for Female Pheromones in Drosophila melanogaster
Source: PLoS One. 2014 Jan 28;9(1):e87509. doi: 10.1371/journal.pone.0087509 (PMC3905024; doi:10.1371/journal.pone.0087509)

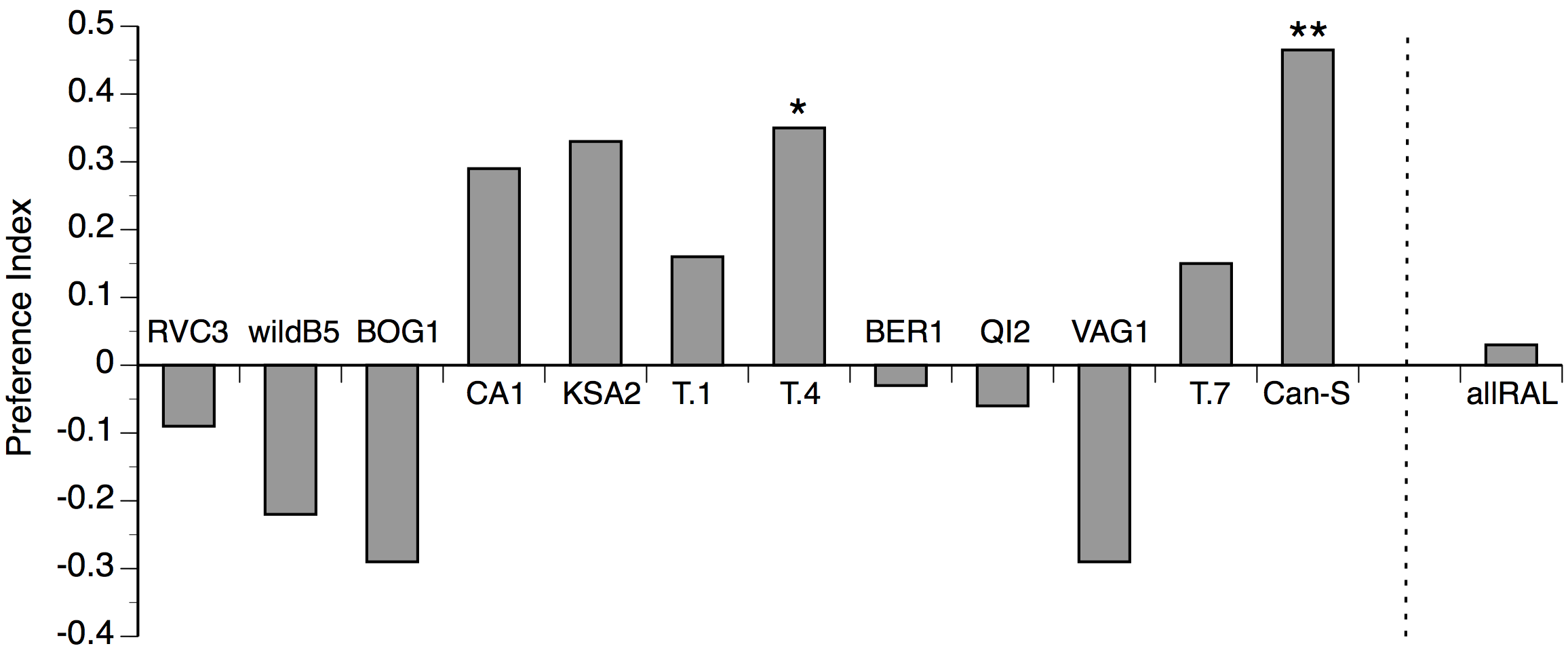

Supplement: Figure S2 — D. melanogaster male preferences when paired with oe− and oe+ females. Preference indices for D. melanogaster males from 12 inbred genotypes and one outbred population (allRAL) when allowed to choose between oe+ females (expressing CHCs) and oe− females (lacking CHCs). The preference index is the relative advantage of oe− females over oe+ females, such that positive values indicate a preference for females lacking CHCs. Asterisks above the columns show preference indices that are significantly different from 0 (binomial tests: * p<0.05; ** p = 0.0001 and significant after sequential Bonferroni adjustment). N (Canton-S) = 71, all other n = 23–54. (TIFF) [file pone.0087509.s002.tiff]
